# Hemolytic uremic syndrome in the setting of COVID-19 successfully treated with complement inhibition therapy: An instructive case report of a previously healthy toddler and review of literature

**DOI:** 10.3389/fped.2023.1092860

**Published:** 2023-02-15

**Authors:** Matija Matošević, Ivanka Kos, Maša Davidović, Maja Ban, Hana Matković, Ivan Jakopčić, Ivana Vuković Brinar, Ágnes Szilágyi, Dorottya Csuka, György Sinkovits, Zoltán Prohászka, Kristina Vrljičak, Lovro Lamot

**Affiliations:** ^1^ Department of Pediatrics, University of Zagreb School of Medicine, Zagreb, Croatia; ^2^ Division of Nephrology, Dialysis and Transplantation, Department of Pediatrics, University Hospital Center Zagreb, Zagreb, Croatia; ^3^ Department of Nephrology, Hypertension, Dialysis and Transplantation, University Hospital Center Zagreb, Zagreb, Croatia; ^4^ Department of Internal Medicine and Haematology, Semmelweis University, Budapest, Hungary; ^5^ Research Group for Immunology and Haematology, Semmelweis University- Eötvös Loránd Research Network (Office for Supported Research Groups), Budapest, Hungary

**Keywords:** hemolytic uremic syndrome, HUS, thrombotic microangiopathy, TMA, COVID-19, complement blockade, ravulizumab

## Abstract

**Introduction:**

As the global pandemic continues, new complications of COVID-19 in pediatric population have turned up, one of them being hemolytic uremic syndrome (HUS), a complement-mediated thrombotic microangiopathy (CM-TMA) characterized by triad of thrombocytopenia, microangiopathic hemolytic anemia and acute kidney injury (AKI). With both multisystem inflammatory syndrome in children (MIS-C) and HUS sharing complement dysregulation as one of the key factors, the aim of this case report is to highlight differences between these two conditions and also emphasize the importance of complement blockade as a treatment modality.

**Case report:**

We describe a 21-month-old toddler who initially presented with fever and confirmed COVID-19. His condition quickly deteriorated and he developed oliguria, accompanied with diarrhea, vomiting and oral intake intolerance. HUS was suspected, supported with compelling laboratory findings, including decreased platelets count and C3 levels, elevated LDH, urea, serum creatinine and sC5b-9 and presence of schistocytes in peripheral blood, negative fecal Shiga toxin and normal ADAMTS13 metalloprotease activity. The patient was given C5 complement blocker Ravulizumab and started to display rapid improvement.

**Conclusion:**

Although reports of HUS in the setting of COVID-19 continue to pour in, the questions of exact mechanism and similarities to MIS-C remain. Our case for the first time accentuates the use of complement blockade as a valuable treatment option in this scenario. We sincerely believe that reporting on HUS as a complication of COVID-19 in children will give rise to improved diagnosis and treatment, as well as better understanding of both of these intricating diseases.

## Introduction

The global pandemic of COVID-19, brought forth by the emergence of SARS-CoV-2, made an impact on our understanding of previously unknown interactions between mechanism of immunological and coagulation cascade in the setting of this peculiar infection. As the pandemic progressed, new conditions and complications, in the form of multisystem inflammatory syndrome in children (MIS-C), arose, oftentimes mimicking other well-known diseases (1, 2). All this made navigating through correct diagnosis and proper treatment a challenge, considering often intertwining clinical presentations and laboratory findings. Despite the fact that clinical manifestations of COVID-19 in children are in general less severe than in adult patients, caution is still warranted considering complications associated with COVID-19 (3, 4). Beside MIS-C, one of these complications might also be thrombotic microangiopathy (TMA), pathological and clinical entity comprised of microangiopathic hemolytic anemia, thrombocytopenia and end-organ involvement. Even though the most common cause of TMA in children is infection with *Shiga* toxin producing *E. coli* (STEC), complement-mediated TMA should not be overlooked, especially considering novel triggers for development of hemolytic uremic syndrome (HUS), a common representative of complement-mediated TMA in children (5–8). Moreover, MIS-C, one of the more serious complications of COVID-19 in children likewise presents with features of TMA paired with complement activity dysregulation (2, 9, 10) (Table 1). This denotes not only a possible diagnostic dilemma, considering the similarities of both HUS and MIS-C, especially when presenting with acute kidney injury (AKI), but also a challenge in timely selection of the most appropriate therapeutic modality. More specifically, MIS-C treatment options comprise mainly of supportive care, IVIG and corticosteroids as the first line and different immunomodulatory drugs (e.g., TNF inhibitor, IL-1 inhibitor, IL-6 inhibitor etc.) as the second-line treatment, while some forms of HUS show excellent response to C5 component inhibitors such as Eculizumab or Ravulizumab (5, 11). Taking into account that complement system plays a key role in both pathogenesis of TMA and COVID-19 (27, 28) and with both HUS and COVID-19 sharing similar pathological findings, novel studies have shown COVID-19 as a potential trigger for HUS, with or without known genetic abnormalities (5, 29–37). With continuation of the pandemic and rise in awareness of COVID-19 as a trigger for HUS, better recognition of COVID-19 and other infectious diseases associated with HUS is expected. As a step in that direction, we describe a compelling case of a 21-month-old toddler with HUS in the setting of COVID-19 successfully treated with complement inhibition. In conjunction with the report, we also conducted a literature review of COVID-19 associated HUS patients with aim to showcase the importance of expeditious diagnosis and treatment of this condition.

**Table 1:** Clinical, laboratory and treatment approach compared between MIS-C and aHUS (2, 5, 6, 9–26).

** **	** **	**MIS-C**	**CM-TMA (aHUS)**
Clinical manifestations	Fever	+++	++
	Abdominal pain	+++	++
	Nausea	++	++
	Vomiting	++	++
	Diarrhea	+	+++
	Cutaneous rash	++	+
	Cardiac damage	+++	++
	Oliguria	++	+++
	Hyper/hypotension	++	+++
	Non-purulent conjunctivitis	+++	−
	Neurological symptoms (e.g., seizures)	++	++
	Respiratory symptoms (e.g., tachypnea)	++	++
Laboratory findings	Thrombocytopenia	++	+++
	Anemia	++	+++
	Elevated LDH	++	+++
	Elevated urea & creatinine	++	+++
	Elevated D-dimers	++	+
	Elevated reticulocytes	+	++
	Elevated CRP & ESR	+++	++
	Reduced C3 serum levels	++	++
	Elevated C5a plasma levels	+	++
	Elevated sC5b-9 plasma levels	++	+++
	Elevated PT & PTT	++	−
	Elevated fibrinogen count	++	−
Other findings	Schistocytes in peripheral bloodsmear	+	+++
	SARS-CoV-2 positivity (serology or PCR)	+++	+
	Positive genetic testing	−	+++
Treatment	Antibiotics (prior to diagnosis)	+++	+
	IVIG	+++	+
	Steroids	+++	+
	Plasma exchange therapy	+	+++
	Anticoagulant therapy	++	+
	Complement inhibition therapy	+	+++
	Other immunomodulatory therapy	++	+

IVIG, intravenous immunoglobulins; PT, prothrombin time; PTT, partial thromboplastin time; CRP, C-reactive protein; ESR, erythrocyte sedimentation rate; LDH, lactate dehydrogenase; −, not reported; +, anecdotal; ++, common; +++, characteristic.

## Methods

A systematic literature search was performed to identify pediatric COVID-19 patients with HUS. The Scopus and MEDLINE/PubMed databases were searched (from November 1, 2019 to October 8, 2022) by entering the keywords “hemolytic uremic syndrome” and “COVID-19” according to the published guidance on narrative reviews. The following parameters were noted from studies including HUS patients: notable findings, treatment, and outcome. Thirty articles describing 44 aHUS patients were found and out of them only nine articles describing 14 pediatric aHUS patients with COVID-19 were found (Supplementary Table S1).

## Case presentation

We describe a case of a 21-month-old toddler who initially presented in February 2022 with a fever of up to 38.5°C and a positive rapid antigen test for COVID-19. His recent medical history included exposure to COVID-19 through the patient’s brother while both parents received two doses of vaccine and showed no relevant symptoms. He was born to non-consanguine parents, with normal birth and growth history and no family history of immune-mediated diseases in any of his relatives. During the second day of illness, he developed watery diarrhea, while on the third day he started to vomit. On the fourth day he became drowse, irritable and intolerant to oral intake. On the fifth day, parents noticed decreased urine output with brown discoloration and decided to take him to an emergency department of a general hospital outside of Zagreb. After performing basic workup, our colleagues who had previous professional and personal experience, suspected HUS and transferred the patient to the emergency department of University Hospital Center Zagreb, where he was admitted to inpatient treatment at Division of Pediatric Nephrology. His initial clinical examination showed normal vital parameters with blood pressure measuring at 109/69 mmHg, pulse at 149/min, oxygen saturation at 98% and axillary temperature at 36.2°C, no signs of dehydration and marginally swollen hands, with urine output under 1 ml/kg/h. Initial laboratory results were indicative of hemolytic uremic syndrome, measuring decrease in red blood cells count (3.32 × 10^12^), hemoglobin (85 g/L) and hematocrit (0.243), along with elevated lactate dehydrogenase (LDH) of up to 3,355 U/L, elevated total bilirubin (46 µmol/L) and normal reticulocytes (57 × 10^9^). Inflammatory markers were slightly elevated, with erythrocyte sedimentation rate measuring 22 mm/h, CRP at 13.6 mg/L and white blood cells count at 17.1 × 10^9^. Platelets count was decreased (19 × 10^9^), while urea, serum creatinine and uric acid were all elevated, measuring 22.4 mmol/L, 204 µmol/L and 659 µmol/L respectively. There was also an increase in cystatin C (4.18 mg/L) as a more specific marker of kidney function. While serum albumin was normal (29.0 g/L), liver enzymes were slightly elevated, measuring 314 U/L for AST, 118 U/L for ALT and 6 U/L for GGT. The patient was marginally acidotic, with pH measuring 7.31 and HCO_3_^−^ measuring at 16 mmol/L. Electrolytes were normal (K^+^ at 4.1 mmol/L and Na^+^ at 137 mmol/L). Urine examination showed elevated proteins in urine (3+), with 341 red blood cells and 5 white blood cells per mm^3^. Moreover, the patient also had nephrotic range proteinuria (3,377 mg/24 h). In addition to aforementioned findings, the patient’s blood smear had a large number of schistocytes. Additionally, there was a noted elevation of D-dimers (7.73 mg/L). Coagulation profile, fibrinogen levels, lipids, immunoglobulins and screenings for cobalamin deficiency and immunological disorders (ANA, ANCA) as well as Coombs test were all normal. Microbiological evaluation was also performed, with positive findings for SARS-CoV-2 (PCR) and nasopharyngeal swab for *Moraxella catarrhalis*. Fecal Shiga toxin, stool and urine culture, as well as throat swab were all negative. Besides, hepatitis B and C serology came back negative and indicating a normal immunization and immunological response (Table 2). Chest x-ray and renal ultrasound were unexceptional. It is worth noting that the patient had no signs of CNS or heart involvement during the whole course of the disease, thus no echocardiography or EEG were performed.

**Table 2:** Initial vital parameters, laboratory, microbiological and imaging findings.

Pathological	Normal
**Laboratory findings**	
ESR (22 mm/h), CRP (13.6 mg/L)	Coagulation profile
WBC (17.1 × 10^9^), RBC (3.32** **× 10^12^)**, Hgb (85 g/L), Htc (0.243), Platelets (19 **× 10^9^), Reticulocytes (57** **×** **10^9^)	Fibrinogen
LDH (3,355** **U/L), Total bilirubin (46** **µmol/L)	Triglycerides
AST (314** **U/L), ALT (118** **U/L), GGT (6** **U/L)	Cholesterol
Urea (22.4 mmol/L), Serum creatinine (204** **µmol/L), Cystatin C (4.18 mg/L), Uric acid (659** **µmol/L)	ANA screen
pH (7.31), HCO_3_^−^ (16** mmol/**L**)**	ENA screen
K^+^ (4.1 **mmol/**L**), Na^+^ (137 mmol/**L**)**	ANCA
Serum albumin (29.0 g/L)	Anti-streptolysin O
D-dimers (7.73 mg/L)	Homocysteine and vitamin B_12_
Urine proteins 3 + **in spot urine, Proteinuria 3**,**377 mg in 24 h urine**	Coombs test
Urine WBC (5/mm^3^), Urine RBC (341/mm^3^)	
Schistocytes in peripheral blood	
**Microbiological findings**	
SARS-CoV-2 PCR positive	Fecal Shiga toxin
Nasopharyngeal swab (positive for *M. catarrhalis*)	Urine culture
** **	Throat swab
** **	Hepatitis B and C serology
**Imaging findings**	
** **	Chest x-ray
** **	Renal ultrasound

Before any therapeutic intervention, a blood sample was sent to our collaborating center for additional testing (Table 3). ADAMTS13 metalloprotease activity was marginally decreased, measuring at 51%, which excluded thrombotic thrombocytopenic purpura (TTP). Haptoglobin was deficient (0.05 g/L) which supported ongoing hemolysis. With normal C4, slight decrease in C3 levels (0.87 g/L) and elevated terminal pathway activation marker sC5b-9 (345 ng/ml), moderate alternative pathway dysregulation was indicated.

**Table 3:** Additional findings of complement pathway testing.

ADAMTS13 metalloprotease activity	51% (67%–150%)
Total complement activity, classical pathway	**49 CH50/ml (48–103 CH50/ml)**
Total complement activity, alternative pathway	**106% (70%–125%)**
Complement C3 level	**0.87 g/L (0.9–1.8 g/L)**
Complement C4 level	**0.18 g/L (0.15–0.55 g/L)**
Factor H antigen level	**411 mg/L (250–880 mg/L)**
Complement factor I antigen	**81% (70%–130%)**
Complement factor B antigen	**153% (70%–130%)**
Anti-factor H IgG autoantibody	**11 AU/ml (<110)**
C1q antigen	**82 mg/L (60–180 mg/L)**
Anti-C1q IgG autoantibody	**1 U/ml (<52)**
sC5b-9 terminal complement complex	**345 ng/ml (110–252 ng/ml)**
Haptoglobin	**0.05 g/L (0.3–2.0 g/ml)**

**Figure 1:**
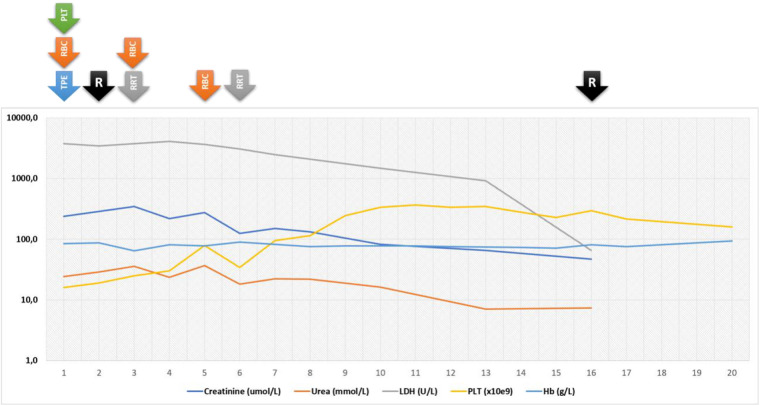
Graphical representation of laboratory values and therapeutical interventions during the course of the disease. R, ravulizumab; TPE, therapeutic plasma exchange; RBC, red blood cells; PLT, platelets.

The initial therapeutic intervention initiated on the first day after admission (sixth day of the disease) consisted of red blood cells and platelets transfusion along with therapeutic plasma exchange (TPE). Due to deteriorating kidney function, oliguria and signs of hypervolemia, renal replacement therapy was performed on days 3 and 6, with additional RBC transfusions administrated on days 3 and 5. With negative microbiology for STEC and *Streptococcus pneumoniae* in addition to complement testing suggestive of alternative pathway dysregulation, HUS in the setting of COVID-19 was suspected. Therefore, on the second day after admission (seventh day of the disease), complement blockade treatment with Ravulizumab, 600 mg i.v. was commenced, with second dose administered after 2 weeks. Interestingly, this led to the progressive amelioration of the patient’s clinical and laboratory findings (Figure 1), so he was discharged from the hospital after 20 days of treatment and two Ravulizumab applications. As per recommendations, along with Ravulizumab he received prophylactic treatment with phenoxymethylpenicillin V as well as vaccination against meningococcal groups A, C, W-135 and Y, and meningococcal group B. After discharge, the patient was regularly followed in our day-care clinic and received two additional doses of Ravulizumab, 6 and 10 weeks after the first administration. Furthermore, the prophylaxis was continued, alongside receiving second doses of before mentioned vaccines. Ten weeks after the initial presentation, his clinical and laboratory findings were completely normal, with no signs of hemolysis, thrombocytopenia nor deteriorated kidney function. More specifically, his CRP was under 1 mg/L, WBC 7.6 × 10^9^, RBC 4.41 × 10^12^, Hb 126 g/L, Htc 0.361, PLT 290 × 10^9^, LDH 275 U/L, and haptoglobin 0.78 g/L. His urea was 4.8 mmol/L, serum creatinine, 25 µmol/L while urine laboratory findings were completely normal (Figure 1). Conclusively, the results of genetic testing, which included multiplex ligation-dependent probe amplification (MLPA) to reveal deletions or duplications in *CFH*, *CFHR1*, *-2*, *-3*, *-4* and *-5* genes, as well as Sanger’s sequencing of the whole coding regions of the complement factor H gene (*CFH*, exon 1–9, 11–23), complement factor I gene (*CFI*, exon 1–13), membrane cofactor protein gene (*CD46*, exon 1–14), complement C3 gene (*C3*, exon 1–41), complement factor B gene (*CFB*, exons 1–18), thrombomodulin gene (*THBD*, exon 1) and complement factor H-related protein 5 gene (*CFHR5*, exons 1–10), were received. No potential disease-causing mutations were identified in the studied genes, although the patient was found to be heterozygous for the *CFH* H3 haplotype (involving the rare alleles of c.-331C > T, Q672Q and E936D polymorphisms), reported as a risk factor of aHUS (38), as well as for the *CFH* V62I polymorphism, that was reported as a protective variant against the development of aHUS (39). Due to outstanding clinical response with sustained remission and previously mentioned genetic results, complement inhibition therapy was suspended, with continuous careful clinical and laboratory monitoring.

## Discussion

To best of our knowledge, this is the first case of HUS in the setting of COVID-19 successfully treated with complement inhibition therapy using Ravulizumab. It is generally known that aside from genetically caused dysregulation of alternative complement pathway, etiological trigger needs to be considered as a factor in development of HUS (6, 40, 41). While *Shiga* toxin producing *E. coli* and in lesser extent *Streptococcus pneumoniae* are most frequently associated with HUS in children, other possible infectious triggers also need to be excluded before considering atypical form of the disease (6). Interestingly, aside from viral infections, predominantly human immunodeficiency virus (HIV) and influenza virus, recent reports from literature point to COVID-19 as a trigger for HUS both in children and adults (Supplementary Table S1) (5, 6, 29, 30). Our patient had positive nasopharyngeal swab for *Moraxella catarrhalis*, a common commensal organism routinely isolated from nasopharyngeal swab in children. In addition, colonization with *M. catarrhalis* can be increased during acute respiratory illness (42–44). Around 0.5 to seven percent of all acute rhinosinusitis are caused by bacterial microorganism, out of which only five to 15 percent are caused by *Moraxella catarrhalis* (45, 46). A paper by van den Heuvel et al. presented a case of *Bordetella pertussis* associated aHUS which also had simultaneous isolation of both *Klebsiella oxytoca* and *Moraxella catarrhalis*, proposing a potential role of those pathogens in the development of HUS, but also stating that exact role of each pathogen in aHUS is not clear (47). Taking into consideration that our patient did not present with signs or symptoms of acute otitis media or acute bacterial rhinosinusitis (48), and that he had COVID-19 infection confirmed by PCR and with signs and symptoms to match, we find it less possible that *Moraxella catarrhalis* could in this particular case be a potential trigger for complement mediated hemolytic uremic syndrome.

The clinical presentation of COVID-19 in children ranges from asymptomatic to mildly symptomatic with fever and cough being the most common symptoms, which suggests that children are as likely as adults to contract SARS-CoV-2, but less likely to present with severe symptoms (49). However, serious complications in children affected by COVID-19 are still possible even after seemingly asymptomatic infection, with most prominent one being MIS-C, but also HUS, as described in this case report (2, 5).

Recent studies on coagulation disorder and vascular pathology in patients with COVID-19 advocate a unique TMA pattern that shares key features with complement-mediated TMA conditions. The proposed mechanism suggests that SARS-CoV-2, after entering cells through ACE2 receptors situated in lungs, endothelial cells and proximal tubules triggers a rapid immune response leading to substantial damage in microvasculature through activation of coagulation cascade and complement system. This thromboinflammatory response further promotes complement activation and thrombotic activity, leading into a vicious cycle of sustained microvascular damage. The basis of complement activation lies in viral S glycoprotein of SARS-CoV-2 which interacts with mannose associated serine protease 2 (MASP2) and mannose binding lectin (MBL), resulting in activation of both lectin and alternative pathways. This results in an increased formation and deposition of membrane attack complex (MAC) on endothelial cells. Furthermore, the interaction between MBL and MASP2 results in a MASP-mediated prothrombin (FII) activation into thrombin (FIIa), which then enters a feedback loop, amplifying its own production *via* coagulation cascade. FIIa also contribute to cleaving of C3 and C5 complement components, adding to proinflammatory and anaphylactic activity. This mechanism can be observed in both COVID-19 patients with TMA and HUS patients (5–7, 50–52).

While complement C5 component blockade with human monoclonal antibodies Eculizumab and Ravulizumab is an established treatment for atypical form of HUS, with better understanding of COVID-19 pathophysiology, especially the role of complement activation, there has been an emerging interest for use of these monoclonal antibodies to reduce immune-mediated consequences of severe infection in COVID-19 patients as well. Supporting evidence for the role of complement, especially C3 activation, in the development of acute kidney injury in COVID-19 came from our recent study (53). Clinical findings of elevated LDH, D-dimer and bilirubin levels, as well as decreased platelet levels paired with anemia, renal injury and diffuse TMA in COVID-19 mimic the pathological findings of HUS, which responds favorably to C5 blockade. Given the role of C5 complement component in perpetuating inflammation and coagulation, as well as induction of a cytokine storm and promoting immune paresis through lymphocyte exhaustion *via* its C5a product, COVID-19 patients may benefit from complement blockade, with studies showing favorable outcomes and significant improvement in patients with severe COVID-19 that receive complement C5 component inhibitor (Supplementary Table S2) (51, 52, 54–56).

A growing body of literature suggest that there is a strong possibility of COVID-19 acting as a trigger for HUS. Nevertheless, it remains to be clarified whether complement activation and the severe endothelial injury, leading to the development of TMA, is a consequence of the cytokine storm typical of MIS-C associated with COVID-19, or if SARS-COV-2 unmasks an underlying primary atypical HUS yet to be confirmed by genetic testing (5). We have previously reported a patient who had hematuria as an early sign of MIS-C, which clearly indicates kidney involvement, although he had no signs of TMA. On the other hand, our current patient could not be classified as having MIS-C, which contributes to the hypothesis that SARS-CoV-2 can trigger HUS in children even in the absence of MIS-C (5, 50). Phospholipase A2 (PLA2G2A) is a proposed proteomic biomarker for development of TMA, as well as MIS-C, in patients with COVID-19 (57).

Although the C3 component was noted to be slightly below referent values in our patient (Table 3), it should be noted that serum C3 level may frequently be normal in the setting of aHUS, while normal level of C4 component might indicate that the patient’s complement evaluation occurred later in the disease course after C4 had normalized and sustained dysregulation of the alternative pathway had been initiated (12, 58). As our patient exhibited many of the clinical symptoms and laboratory signs, including complement abnormalities, pointing towards atypical form of HUS (aHUS), the best way to classify his disease would be as HUS triggered by viral infection, in this case COVID-19. Although the confirmation of this diagnosis is impossible without the genetic testing, a high level of suspicion supported with available clinical and laboratory findings should prompt the treatment, as described in our case, since timely administration of complement blockade is crucial for its success, regardless of the fact that in outlined setting TMA seems to be epiphenomenon (6, 41). Nevertheless, before reaching this complex decision, we used “conventional” modalities, such as TPE, until STEC-HUS and TTP could be excluded. Moreover, our approach differed in the selection of complement blockade agent, with our preferences set to Ravulizumab, which to the best of our knowledge was not used before in this setting. This decision was supported by its 4-fold longer half-life in comparison to Eculizumab, therefore requiring less frequent maintenance doses and leading to reduced infusion burden, which is desirable in pediatric patients (43). Although our patient received this treatment for less than 3 months, it was just enough to achieve sustained remission and make an informed decision to discontinue the treatment after the results of genetic testing showed no pathogenic or likely pathogenic rare variant. Interestingly, our patient is heterozygous carrier of the *CFH* H3 haplotype reported as a risk factor for complement mediated HUS. This risk haplotype alone might never be considered as a pathogenic factor for the development of aHUS, but within the state of turmoil elicited by COVID-19, the full-blown disease emerged. This two-hit hypothesis might be further supported by the fact that after the initiation of complement blockade and resolution of COVID-19, everything returned to normal, and maintained as such even after cessation of treatment. Nevertheless, since this happened during the first years of patient’s life, it remains to be seen if other “hits” will cause any new TMA episodes.

As a conclusion, the presented case suggests that patients showing symptoms and signs of HUS in the setting of COVID-19 deserve detailed evaluation for activity markers of TMA, as well as potentially predisposing genetic intrinsic factors. In such patients, early clinical recognition with specific testing is crucial for treatment decisions and long-term management, while detailed descriptive studies may fuel future research on pathogenesis and new treatment options. Finally, we consider our case report a worthy contribution to a growing body of literature aimed at better understanding of an intricate relationship between TMA and COVID-19, as well as a worthy example illustrating that short course of complement blockade treatment might be beneficial for COVID-19 patients with immunothrombosis.

## Data Availability

The original contributions presented in the study are included in the article/**Supplementary Material**, further inquiries can be directed to the corresponding author.
