# Supplementary material for: Hemolytic uremic syndrome in the setting of COVID-19 successfully treated with complement inhibition therapy: An instructive case report of a previously healthy toddler and review of literature
Source: Front Pediatr. 2023 Feb 15;11:1092860. doi: 10.3389/fped.2023.1092860 (PMC9975343; doi:10.3389/fped.2023.1092860)
Supplement: Supplementary file 1 [file Table1.docx]

**SUPPLEMENTARY TABLE 1**

Supplementary Table 1. Notable findings, treatment modalities and outcome of aHUS pediatric patients with COVID-19

| First author (ref. no.) | No. Of Pts. | Pt Age | Notable findings | Treatment | Outcome |
| --- | --- | --- | --- | --- | --- |
| Alizadeh (14) | 1 | 16 months | Reticulocytosis 13%  Undetectable haptoglobin  Elevated LDH (peak 3.190 u/L)  Hiperbilirubinemia (peak 1.5 mg/dL)  Schistocytes in peripheral blood smear  Macrothrombocytopenia  Rising BUN & creatinine (peak at 39 mg/dL and 0.39 mg/dL)  Low CH50 (3 units), elevated CFH, CFI, Bb fragment level and sMAC  Normal serum C3 and C4  Nephrotic-range proteinuria | Eculizumab  Antihypertensives | Anemia and thrombocytopenia improved, discharged with eculizumab maintenance every 3 weeks |
| Mahajan (15) | 1 | 14 years | Creatinine 8.97 mg/dL  BUN 170 mg/dL  Proteinuria 100-300 mg/dL  Thrombocytopenia (126 000/mL)  Hgb 6.8 g/dL  Elevated LDH 4087 U/L  Elevated bilirubin 6.2 mg/dL  Schistocytes in peripheral blood smear  Low serum C3 33 mg/dL  Low serum C4 4.0 mg/dL  High C5b9 level 1669 ng/L | Eculizumab  IV pulse steroids  IVIG  Anakinra  Antihypertensives  CRRT | Renal function stabilized after 3 weeks; discharged |
| Dalkıran (16) | 1 | 3 years | Creatinine 1.9 mg/dL  Anuria  Progressive thrombocytopenia  Microangiopathic hemolytic anemia  Undetectable haptoglobin  Reticulocytosis 8%  LDH 2540 U/L  Schistocytes in peripheral blood smear | Plasmapheresis  Antibiotics  Antihypertensives  IVIG | Completely asymptomatic after 4 weeks; discharged with oral antihypertensive treatment |
| Hamza (17) | 1 | 11 years | Hgb 6.8 g/dL  Reticulocytes 5.5%  BUN 107 mg/dL  Creatinine 6.3 mg/dL  Total bilirubin 7.6 mg/dL  Urine protein ++  Schistocytes in peripheral blood smear | Plasma transfusion  Hemodialysis | Admitted to ICU; unknow outcome of aHUS |
| Nomura (18) | 1 | 9 years | Hypertensive (136/86 mmHg)  Hgb 9.3 g/dL  Thrombocytopenia 148 x 10^3^ U/L  Elevated LDH 1835 U/L  BUN 256 mg/dL  Creatinine 20.9 mg/dL  Hematuria, nephrotic-range proteinuria  Schistocytes in peripheral blood smear | Inpatient hemodialysis  Supportive care  Antihypertensives | Kidney function & proteinuria improved at 6 week follow-up |
| Khandelwal (19) | 5 | 4, 12, 13, 7 and 10 years | Decreased Hgb  Elevated LDH  Thrombocytopenia  Decreased serum C3  Positive anti-CFH antibodies | IV cyclophosphamide (pts #1 and 5)  Prednisone (pts #1-5)  Mycophenolate mofetil (pts #2-4) | Kidney function recovery 2-7 days (pts #1-4) and 12 days (pt #5) after hemodialysis; anti-CFH antibodies in median range at 4 months follow-up |
| Van Quekelberghe (20) | 2 | 4 and 4.5 months | Anemia  Thrombocytopenia  Elevated LDH  Schistocytes in peripheral blood smear  Hypertension  AKI with anuria & elevated creatinine (pt #2)  Swollen, hyperechogenic kidneys with reduced perfusion (pt #2)  Slightly elevated sC5b-9 (456 ng/mL for pt #1 and 311 ng/mL for pt #2) | Eculizumab  Hemodialysis  Antibiotics (pt #1)  Antihypertensives | Discharged; hypertension & persistent proteinuria present five months afterd discharge |
| Marcilla Vasquez (38) | 1 | 5 years | Anemia  Thrombocytopenia  Elevated LDH  2% schistocytes in peripheral blood smear  Haptoglobin < 3 mg/dL  Moderate proteinuria | N/A | Due to clinical improvement, treatment for aHUS was not started; outcome remains unknown |
| Emami (39) | 1 | 11 years | Hemolytic anemia  Thrombocytopenia  High serum creatinine  Proteinuria without hypoalbuminemia | Plasmapheresis | Discharged in good condition; serum creatinine remained at 2 mg/dL, proteinuria without hypoalbuminemia continued |
