# Supplementary material for: Hemolytic uremic syndrome in the setting of COVID-19 successfully treated with complement inhibition therapy: An instructive case report of a previously healthy toddler and review of literature
Source: Front Pediatr. 2023 Feb 15;11:1092860. doi: 10.3389/fped.2023.1092860 (PMC9975343; doi:10.3389/fped.2023.1092860)
Supplement: Supplementary file 2 [file Table2.docx]

**SUPPLEMENTARY TABLE 2**

Supplementary Table 2. Notable publications describing usage of C5 inhibitors in COVID-19 patients

| First author (ref. no.) | Publication type | No. of patients | Choice of treatment | Outcome |
| --- | --- | --- | --- | --- |
| Mastellos (52) | Exploratory study | 10 | Eculizumab | 2 pts died, 8 pts showed improvement, were discharged |
| Ruggenenti (53) | Single-center, academic, unblinded study | 10 | Eculizumab | Improved respiratory function, lower mortality and chronic complications after discharge |
| Annane (54) | Nonrandomized, controlled study (proof-of-concept) | 80 | Eculizumab | 35 pts recieved eculizumab; greater estimated survival on day 15 with eculizumab, significant decrease in LDH, BUN and bilirubin; rapid increase in platelets count, PT; reduced hypoxia and inflammation |
| Laurence (55) | Case report | 3 | Eculizumab | Improved laboratory results, normalization in liver function & creatinine, complete remission of AKI & heart failure in one pt, partial remission in others; one pt died of respiratory failure |
| Raghunandan (56) | Case report | 1 | Eculizumab | Pt was on CVVH; improved respiratory function after 2 days, extubated after 20 and weaned off CVVH by 22nd day; improvement in hypertension; undergoing rehabilitation |
| Pitts (57) | Experimental immunosuppressive therapy trial | 11, 5 recieved treatment | Eculizumab | 3 pts died due to COVID-19 complications; 2 pts showed daily improvement after recieving treatment, were extubated and discharged |
| de Latour (58) | Letter to the Editor | 8 | Eculizumab | 6 pts showed significant improvement in respiratory functions; 2 pts died due to COVID-19 complications |
| Burwick (59) | Case series | 8 | Eculizumab | 6 pts were pregnant, 2 were postpartum; following treatment inflammation markers were decreased |
| Giudice (60) | Brief research report | 17 | Eculizumab + ruxolitinib (7 pts) | Showed decreased D-dimer levels and significantly increased platelet count; improved CT scan imaging;  no secondary infections; lower incidence of lymphopenia |
| Diurno (61) | Case series | 4 | Eculizumab | All pts showed a significant clinical improvement within the first 48h; all patients sucessfully recovered |
