# Supplementary figures and images for: Hemolytic uremic syndrome in the setting of COVID-19 successfully treated with complement inhibition therapy: An instructive case report of a previously healthy toddler and review of literature
Source: Front Pediatr. 2023 Feb 15;11:1092860. doi: 10.3389/fped.2023.1092860 (PMC9975343; doi:10.3389/fped.2023.1092860)

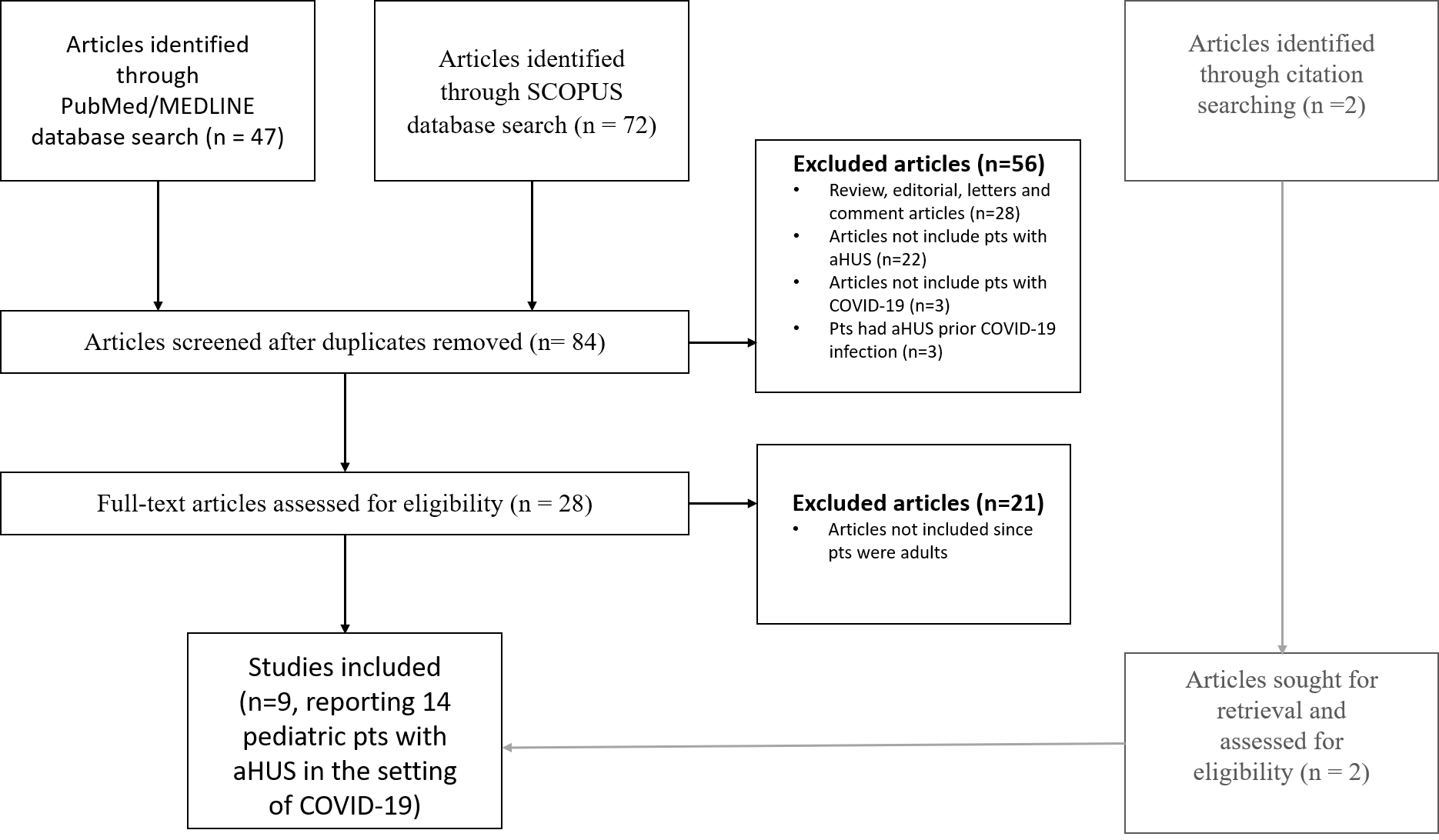

Supplement: Supplementary file 3 [file Image1.tif]
